# Focus on clinical workflows

**DOI:** 10.1002/acm2.12753

**Published:** 2019-10-21

**Authors:** Per Halvorsen

**Affiliations:** ^1^ Radiation Oncology Lahey Health Burlington MA USA

After more than a decade of work and internal review and deliberation, the AAPM’s Task Group 100 report was published in the journal *Medical Physics* in July 2016.^1^ The report constitutes the AAPM’s endorsement of a paradigm shift in the approach to quality management in radiation oncology.

The task group’s original charge was to develop an update to the TG‐40 report on radiotherapy quality management. Drs. Saiful Huq and Bruce Thomadsen have described to me that as the task group members assessed the state of radiotherapy circa 2005, they concluded that the radiotherapy process had evolved significantly in recent decades with much more information feeding into a more‐complex decision process and the attendant complexity of hand‐offs between team members. As the authors state in the report, the traditional quality management approach placed a strong emphasis on quality control of equipment, and while this has served the profession well and will continue to serve an important role, the risks from the clinical process may be a more significant factor in modern radiotherapy. They concluded that the profession needs better tools to assess and understand the risks in each clinic’s unique process.

Data from RO‐ILS support the TG‐100 conclusion. The RO‐ILS database now contains >10,000 events, providing a critically important view of failure‐mode trends in the profession. The step in the radiotherapy process with the highest rate of reported incidents is treatment planning with fully 30% of all reported events, followed by the treatment delivery process with 26% of all reported events.^2^ Some of the most common failure modes in treatment planning include hand‐off between team members, contouring of targets and regions of interest, ambiguous physician instructions, treatment plan revisions, and rushed planning timelines. In treatment delivery, some of the common failure modes include patient identification, isocenter alignment, and HDR brachytherapy treatments with incorrect source guide tube length or applicator diameter. None of the aforementioned failure modes would have been prevented by equipment quality control alone. Clearly, a focus on the clinical process is critically important if we wish to mitigate these demonstrated risks in modern radiotherapy.

Yet, much of the early focus on TG‐100 implementation has been to use the TG‐100 methodology to critique existing equipment quality control recommendations. This bias is understandable — equipment quality control is our “comfort zone” as medical physicists, and applying the new methodology to something familiar is perhaps one way to gain confidence in the new methodology. **I posit that we’re missing a significant opportunity if we restrict our efforts to this narrow scope**. Doing so would limit the risk analysis approach to the physics team, missing the opportunity to engage the entire clinical team in better understanding their process. Any suggested prioritization would collide with concerns about local regulatory requirements or accreditation standards for equipment quality control, with a likely outcome being that nothing changes and the effort feels futile. By contrast, the clinical workflows are highly variable and without prescriptive national standards, and affect the entire radiation oncology clinical team from the receptionists to nurses, dosimetrists, radiation oncologists, radiation therapists — and medical physicists. With our clinical training and strong analytical skills, medical physicists can add significant value to their institutions by helping the clinical team to optimize their workflows for safety and efficiency. In the process, the stature of the medical physics profession would be further elevated.

*So what’s holding us back?* Several negative dynamics. The TG‐100 project, while an admirable and enormous effort, was not designed as a collaborative effort with our physician, radiation therapist and dosimetrist sister societies. That’s a missed opportunity for which we continue to pay a price in terms of poor awareness and lackluster engagement. More fundamentally, and this is sad to acknowledge, many (if not most) healthcare organizations have not yet been effective in adopting a truly pervasive organizational safety culture. Most organizations claim they’re in favor of a robust safety culture and may even believe they’ve succeeded in that regard, but my own experience from 30 years in the clinic and from numerous accreditation surveys is that most organizations are loath to tackle workflow inefficiencies and weaknesses that originate with the physicians or with administrative edicts. I recall a recent episode whereby the chief physicist and department manager took the initiative to invite staff from the organization’s process‐improvement office to conduct a “LEAN analysis” of the radiation oncology workflow — only to be chastised by the administration and physicians for “making them look bad”. This occurred in an organization that has officially endorsed the “High‐Reliability Organization” principles.

The TG‐100 report recommends the adoption of three mature risk analysis tools toward risk‐informed development of radiotherapy quality management programs: process mapping, failure modes and effects analysis, and fault tree analysis. Together, these tools, when properly adapted to radiation oncology, provide a framework for identifying relative risks in each clinic’s radiation oncology program. But they are really just tools for doing what any robust safety culture would do: Critically assess the process to optimize the likelihood of a high‐quality outcome, and design interventions to mitigate the weakest steps in the process regardless of who it originates with. Process failures are not theoretical; there are many examples of serious process failures in our disciplines (both imaging and therapy) that have resulted in patient harm and, in some cases, death. Less than a decade ago, our then‐President Mike Herman was called to testify to the US Congress about such process failures in our field; who among us genuinely believes that such risks have been eliminated?

Medical physicists can play a leadership role by coaching their clinical team through the learning curve of adapting these tools to their work environment, first and foremost with a focus on the clinical workflows. The result would be a more robust practice that is well‐positioned to safely adopt new treatment approaches and technologies into their clinic, with a stronger safety culture facilitated by the team approach inherent in risk‐informed quality management. As a clinical medical physicist, I can think of no more compelling motivation.

At a recent training workshop, I was asked how to confront organizational culture or physician resistance, and how would one know when they’ve been successful in implementing a robust safety culture. To the former, I would say “head on” — most organizations officially endorse safety culture principles, so call their bluff. As long as you’re conducting thoughtful risk analysis of the clinic’s processes and sharing the information with the clear goal of mitigating those risks, you’re on high ground and exhibiting professional leadership. To the latter, I would say you’ve succeeded when critical process analysis and open, candid dialogue about the findings becomes a natural component of the clinical team’s approach to their work. When this occurs organically, without constant prompting by the medical physicist, you have arrived. {Full disclosure: I haven’t arrived yet, but the destination no longer seems out of reach.}
